# The Immunomodulatory Activity of *Punica granatum* L. Peel Extract Increases the Lifespan of Mice with Lethal Sepsis

**DOI:** 10.1155/2023/2868707

**Published:** 2023-08-16

**Authors:** Liana de O. Trovão, Liliane dos S. Rodrigues, Priscila M. Mendes, Patrícia C. S. Alves, Aluísio da S. Oliveira, Jefferson M. Brito, André A. M. Vale, Dimitrius V. de O. Garbis, Gisele Simão, Ana Paula S. A. dos Santos, Paulo V. S. Pereira, Lucilene A. Silva, Andresa A. Berretta, Flávia R. F. Nascimento, Rosane N. M. Guerra, Valério Monteiro-Neto, Elizabeth S. Fernandes, Márcia C. G. Maciel

**Affiliations:** ^1^Federal University of São Paulo, São Paulo, Brazil; ^2^Federal University of Maranhão, São Luís, Brazil; ^3^Programa de Pós-graduação em Biotecnologia Aplicada à Saúde da Criança e do Adolescente, Faculdades Pequeno Príncipe e Instituto de Pesquisa Pelé Pequeno Príncipe, Curitiba, Brazil; ^4^Apis Flora Indl. Coml. Ltda., São Paulo, Brazil; ^5^University of Brasília, Brasília, Brazil

## Abstract

Sepsis is an organ dysfunction syndrome associated with high mortality. To date, no effective treatment is available to combat this disease. *Punica granatum* L. is a potential alternative treatment due to its anti-inflammatory, antimicrobial, and antioxidant properties. Thus, this study aimed to evaluate the effects of a hydroalcoholic crude extract from the peels of *P. granatum* (HCEPg) in mice with lethal sepsis. Lethal polymicrobial sepsis was induced in female Swiss mice via cecal ligation and puncture (CLP). Initially, the animals were divided into three groups: Sham (false-operated), CLP-control (phosphate-buffered saline), and CLP-HCEPg (single dose, 5 mg/kg, subcutaneous administration). Treatment was initiated immediately after the induction of sepsis, and survival was evaluated every 12 hr for 5 days. Those who survived were euthanized. Serum cytokine levels were measured using a cytometric bead array Mouse Inflammatory Cytokine Kit. The number of colony-forming units, as well as the number of cells in the lymphoid organs and their activation markers, were analyzed. Results showed that treatment with HCEPg increased lifespan and reduced bacterial counts in the peritoneum, bloodstream, and spleen. HCEPg also decreased hydrogen peroxide secretion by phagocytes and augmented serum IL-10 levels, indicating its systemic anti-inflammatory effects. Additionally, treatment with HCEPg attenuated infection-induced lung hemorrhage. Overall, *P. granatum* extract improved the lifespan of septic mice, possibly due to its antimicrobial, anti-inflammatory, and immunomodulatory effects, thereby regulating bacterial load and translocation, as well as controlling the systemic inflammation induced by sepsis.

## 1. Introduction

Sepsis is a severe public health problem and the leading cause of death in intensive care units, mainly among the elderly (80 years and older) and immunosuppressed patients [1]. It is an inflammatory syndrome with high mortality, with nearly 30 million cases per year worldwide, potentially leading to 6 million deaths [2, 3]. It comprises different stages, including septic shock, in which endotoxins and exotoxins activate endothelial cells and leukocytes that significantly increase the production of inflammatory mediators, resulting in generalized inflammation associated with infection [4, 5].

The manipulation of pathways that modulate inflammation by targeting complex interactions between early and late inflammatory mediators may offer a novel approach to markedly improve the mechanistic understanding of sepsis and the development of clinical therapies [6]. Several studies have focused on the discovery of novel therapeutic agents for sepsis, including thrombomodulins, immunoglobulins, corticosteroids, vasopressors, and endogenous enzymes [7]. In this context, cytokines and chemokines are essential mediators of sepsis, playing roles in both the inflammatory and anti-inflammatory phases of the syndrome. Their production, mostly by inflammatory cells, regulates tissue damage, and endothelial dysfunction, ultimately contributing to organ failure and vascular collapse or to tissue recovery. Their roles are complex, meaning that targeting them has proven to be a challenge. These aspects have been extensively reviewed and recently discussed [8–10]. However, immunomodulatory therapy is directed and restricted to the treatment of persistently immunosuppressed patients and is not fully effective alone. In addition, antibiotic administration is not ideal since prolonged and unnecessary antibiotic use can lead to antimicrobial resistance [11].

There is an increasing search for biologically active substances with antimicrobial and immunomodulatory properties for the treatment of inflammatory disorders, such as sepsis. Interestingly, medicinal plants could serve as an alternative treatment for sepsis-related complications in critically ill patients [12]. Makled et al. [13] orally administered pomegranate fruit extract 2 weeks before sepsis induction in rats. They described its anti-inflammatory and antioxidant properties, and its protective effect against acute liver injury in rats, improving survival. Thus, we aimed to investigate the potential of a single dose of *Punica granatum* peel extract in increasing the survival of septic mice.

Pomegranate (*P. granatum* L.), which belongs to the Punicaceae family, is a medicinal plant widely distributed in Brazil, where it is popularly known as “romã,” “pomegranato,” or “granado” [14]. Its leaves, stem bark, fruits, and flowers have been used to elucidate its various ethnopharmacological applications for the treatment of bacterial, fungal, and virus infections; fever, oral inflammatory diseases, bronchitis, hemorrhoids, skin and mucosal abscesses, and conjunctivitis, among others [14–17].

An extensive list of compounds is found in various plant parts. The main compounds include anthocyanins (present in the fruit juice and pericarp), ellagic acid (EA; fruit juice, peel, and flowers), punicalin and punicalagin (pericarp, leaves, and fruit peel), and flavonoids (pericarp and leaves) [14]. Gallic acid, EA, and punicalagin are associated with the antimicrobial effects of *P. granatum* [18]. *P. granatum* extract was found to have a satisfactory potential against *Staphylococcus aureus* and methicillin-resistant, demonstrating its use in the treatment of serious infections [19, 20]. The extract also inhibited several microorganisms, including those causing diarrhea (*Escherichia coli*, *Salmonella paratyphi*, *Salmonella typhimurium*, *Salmonella infantile*, *Salmonella enterica*, *Salmonella brunei*, *Vibrio parahaemolyticus*, *Yersinia enterocolitica*), wounds (*Proteus mirabilis* and *Pseudomonas aeruginosa*), respiratory infections (*Klebsiella pneumoniae* and *Haemophilus parahaemolyticus*), and skin infections (*Staphylococcus epidermidis* and *Candida albicans*) [21]. Its antioxidant and anti-inflammatory properties are associated with the presence of punicaline, punicalagin, and pedunculagin [16, 17, 22]. In addition, the hexane fraction of *P. granatum* leaves at a dose of 100 mg/kg did not maintain the survival of septic mice but reduced the production of IL-6, nitric oxide, and hydrogen peroxide in the peritoneal lavage cells [23]. All these activities are essential to treat syndromes involving an imbalance in the inflammatory response caused by a disseminated infection, such as sepsis.

Based on the antimicrobial and anti-inflammatory potential of *P. granatum*, the aim of this study was to investigate the effect of subcutaneous treatment with a single dose of *P. granatum* extract on the control of bacterial growth and systemic inflammatory responses in a lethal murine model of sepsis induced by CLP.

## 2. Materials and Methods

### 2.1. Animals

Female, 8–12-week-old Swiss mice (average weight: 25 g) were used in this study. Animals were obtained from the Biological Service Unit of the Universidade Federal do Maranhão (São Luiz, Brazil), where they were maintained at 26 ± 2°C and 44%–56% relative humidity under a 12 hr light–dark cycle and with free access to sterile food and water. The animals were kept in cages with dimensions 41 × 34 × 18 cm^3^ (length × depth × height) with a removable grid. The study protocol was approved by the Animal Use Ethics Committee of the Universidade Federal do Maranhão (Protocol: 23115.002346/2005-45). All surgeries were performed under anesthesia, and efforts were made to minimize suffering.

### 2.2. Preparation of the Fruit Peel Extract

The hydroalcoholic crude extract from the fruit peel of *P. granatum* (HCEPg) was supplied by Apis Flora® and maintained at room temperature until use. The HCEPg was concentrated under reduced pressure, dried in an oven at 37°C, and stored at 4°C in sterile flasks until use. A lyophilized dry residue diluted in isotonic phosphate-buffered saline (PBS) was used in the succeeding experiments.

### 2.3. Chemical Characterization of Fruit Peel Extract

To determine the total phenolic content, an analytical curve for tannic acid (Sigma–Aldrich) was generated. Pomegranate extract was prepared in a 50 mL volumetric flask using water as solvent. The samples were homogenized, and the flasks were then placed in an ultrasonic bath for 30 min. A 0.5 mL aliquot was transferred to another 50 mL flask with 2.5 mL of Folin–Denis reagent and 5.0 mL of 29% sodium carbonate. The samples were protected from light, and absorbance at 760 nm was measured after 30 min using a UV–VIS spectrophotometer. All samples were analyzed in triplicates.

EA was obtained from Fluka (95.0%, Batch BCBN4398V). High-performance liquid chromatography (HPLC)-grade methanol was supplied by J.T. Baker (Mexico City, Mexico), and purified water was obtained using a Milli-*Q* Direct Q-5 filter system (Millipore, Bedford, USA). Analytical-grade acetic acid was purchased from Synth (Labsynth, Diadema, Brazil). To determine EA content (EAC), the extracts previously diluted in methanol were vortexed and incubated for 30 min in an ultrasound bath. The solution was filtered and subjected to HPLC analysis (Shimadzu apparatus equipped with a CBM controller, LC-20AT quaternary pump, SPD-M 20 A diode-array detector, and autosampler, Shimadzu LC solution software, version 1.21 SP1) using a 100 × 2.6 mm Shim pack ODS C18 column. The mobile phases used were methanol and acetic acid aqueous solution 2% along an elution gradient (20%–72.5% v/v methanol for 0–7 min, 72.5%–95% v/v methanol for 7–7.5 min, 95% v/v methanol for 7.5–8.5 min, 95%–20% v/v methanol for 8.5–9 min, and 20% v/v methanol for 9–10 min), with a flow rate of 1.0 mL/min and an oven temperature of 25°C. The eluted samples were analyzed using a UV detector at 254 nm. A calibration curve was constructed by plotting the peak area (*y*) against concentration in *μ*g/mL of standard solutions (*x*). The standard equation obtained from the curve was used to quantify EA (mg/g of sample extract). All assays were performed in triplicate.

### 2.4. Induction of Sepsis

Polymicrobial sepsis was induced using the cecal ligation and puncture method described by Benjamim et al. [24], with minor modifications. Briefly, all mice were intraperitoneally anesthetized with a mixture of 100 mg/kg ketamine hydrochloride and 10 mg/kg xylazine hydrochloride. Laparotomy was performed, and the cecum was mobilized, ligated below the cecal valve, and punctured 10 times using an 18-gauge needle to induce lethal sepsis [25]. The cecum was placed back into the peritoneal cavity, and the abdomen was closed in two layers. Saline (0.5 mL/10 g body weight) was administered subcutaneously to the animals as a hydration fluid.

### 2.5. Experimental Design

A total of five mice was randomly assigned to each experimental group: the Sham group, wherein mice were anesthetized and underwent laparotomy surgery without cecal ligation and puncture; the CLP group, wherein mice cecum was ligated and punctured; and the HCEPg group, wherein a single dose of HCEPg (5 mg/kg) was subcutaneously administered immediately after sepsis induction. Two in vivo experiments were performed: (1) survival analysis and (2) evaluation of cellular/immunological/functional parameters. Biological samples were collected 12 hr after CLP when the mice were euthanized with an overdose of anesthetic (150 mg/kg ketamine hydrochloride and 120 mg/kg xylazine hydrochloride) [26]. Blood and cells were collected to evaluate the antimicrobial, anti-inflammatory, and immunological properties [25]. To evaluate lifespan, the number of remaining animals was recorded every 12 hr until the 5th day of observation and euthanized as described previously [27].

### 2.6. Spleen, Bone Marrow, and Lymph Node Cell Counting

After euthanasia, the femur, spleen, and mesenteric lymph nodes were excised. The femur was perfused with 1 mL of PBS to isolate the bone marrow cells. The mesenteric lymph node was triturated with 1 mL of PBS, while the spleen was washed with 5 mL of PBS and passed through a silk sieve. The cells were stained with 0.05% crystal violet (in 30% acetic acid) and counted using a bright-line Neubauer chamber (Sigma–Aldrich) under a standard light microscope at 400× magnification.

### 2.7. Peritoneal Cell Collection and Counting

Peritoneal cells were harvested by washing the peritoneal cavity with 5 mL of sterile ice-cold PBS. Cells were stained with 0.05% crystal violet (in 30% acetic acid) and counted using a bright-line Neubauer chamber (Sigma–Aldrich) under a standard light microscope at 400× magnification. Differential cell counts were performed using CytoSpin preparations stained with Instant-Prov (NewProv, Pinhais, Brazil) [28].

### 2.8. Histological Analysis

The lungs were extracted, fixed in 10% formalin, and embedded in paraffin. Next, 5 *μ*m thick tissue sections were cut using a microtome, mounted on slides, and stained with hematoxylin and eosin. The slides were examined under a light microscope at 20×, 40×, and 100× objectives. Hemorrhage, inflammatory infiltrates, necrosis, and edema were analyzed. Tissue changes were scored as follows: 0, absent; 1, scarce; 2, moderate; and 3, intense [29].

### 2.9. Colony-Forming Unit (CFU) Determination

Briefly, 10 *μ*L of the blood, peritoneal lavage, and cell suspension of the lavage obtained from the spleen macerate were collected for bacterial quantification. Each sample was serially diluted (blood, 10^4^ CFU/mL; peritoneum, 10^8^ CFU/mL; spleen, 10^1^ CFU/mL) in 90 *μ*L of PBS in each well of a 96-well plate. Aliquots (100 *μ*L) were then plated on Brain Heart Infusion agar plates (Difco Laboratories, Detroit) and seeded using a Drigalski handle. CFUs were counted after overnight incubation at 37°C. The results are expressed as CFU/mL [25].

### 2.10. Hydrogen Peroxide (H_2_O_2_) Production

H_2_O_2_ production was assessed using the dihydrorhodamine 123 (DHR) assay (Sigma®). Briefly, cell suspension from the peritoneal cavity was counted and adjusted to a concentration of 1 × 10^6^/mL. The cells were labeled with DHR (Sigma®) (375 ng/mL) for 10 min at 37°C and stimulated with phorbol 12-myristate 13-acetate (PMA, Sigma®) (50 nM) for 1 hr at 37°C. Subsequently, the cells were placed on ice for 10 min to terminate the reaction. The cell suspensions were washed with PBS, centrifuged at 4°C, and analyzed using a flow cytometer [26, 30].

### 2.11. Flow Cytometry

Cells from the peritoneal cavity, mesenteric lymph nodes, and spleen were adjusted to 10^6^ cells/mL, transferred to a 96-well plate, and incubated with specific antibodies for 15 min. Following incubation, the cells were fixed with 1% paraformaldehyde (Fisher, Waltham, MA, USA) and analyzed using a flow cytometer (Guava®). A total of 10,000 events were acquired, and cell size (forward scatter, FSC) and complexity (side scatter, SSC) were used to establish an acquisition gate, followed by fluorescence channel analysis. Subsequently, the data were processed using the FlowJo v10.0 software (Tree Star Inc.). Up to 1% of false-positive events were observed in isotype control samples. The results were expressed as mean fluorescence intensity (MFI).

### 2.12. Cytokines Quantification

The cytometric bead array (CBA-Mouse Inflammation Kit-BD™ Cytometric Bead Array-Cat no. 552364) technique was performed to quantify tumor necrosis factor (TNF)-*α*, monocyte chemoattractant protein-1 (MCP-1), interleukin (IL)-6, IL-10, IL-12, and interferon (IFN)-*γ* in the serum, as described previously [31] using a mouse inflammatory cytokine kit (Becton Dickinson Biosciences, San Jose, CA, USA).

### 2.13. Statistical Analysis

The results are expressed as means. The normality of the data was evaluated using the D'Agostino–Pearson test. Statistical analysis was performed using ANOVA (post hoc tests: Newman–Keuls) using the GraphPad Prism software version 5.0. Differences were considered statistically significant at *p* < 0.05. Kaplan–Meier curves were used for survival analysis, and the log-rank test was used to compare the curves. All experiments were repeated at least twice.

## 3. Results

### 3.1. Chemical Characterization of HCEPg

**Figure 1:**
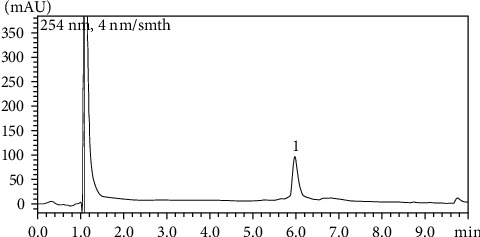
Chemical profile of HCEPg using HPLC. Ellagic acid (EA) and total phenolic content, including tannic acid. The mobile phases used for EA were methanol and acetic acid aqueous solution (elution gradient of 20%–72.5% v/v methanol for 0–7 min, 72.5%–95% v/v methanol for 7–7.5 min, 95% v/v methanol for 7.5–8.5 min, 95%–20% v/v methanol for 8.5–9 min, and 20% v/v methanol for 9–10 min). The flow rate was 1.0 mL/min, and the oven temperature was set at 25°C. A 100 × 2.6 mm Shim pack ODS C18 column was used, and the eluted samples were detected at 254 nm using a UV detector.

HCEPg contained 6.34 mg/g of EA and 0.83 mg/g of total phenolic compounds, such as tannic acid. Considering that 5 mg of pomegranate powder was administered per kg of mice, 4.15 and 0.0317 mg were estimated to correspond to total phenolic compounds and EA, respectively. The HPLC chromatogram was obtained by focusing on EAC (Figure 1).

### 3.2. HCEPg Reduces Sepsis Mortality

**Figure 2:**
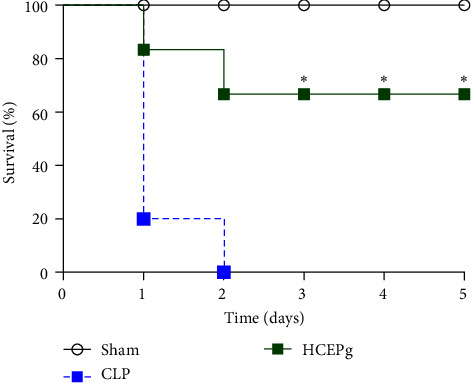
*P. granatum* extract (HCEPg) increased the survival of mice with sepsis. Mice were divided into three groups: Sham (untreated Swiss mice without sepsis), control (CLP), and HCEPg (animals treated subcutaneously with a single dose of HCEPg (5 mg/kg) immediately after induction of sepsis). The survival was monitored for 5 days, and the death rate was recorded every 12 hr. ( ^*∗*^) *p* < 0.05 compared to the CLP group.

All mice in the control (CLP) group (100%) died 2 days after lethal sepsis induction, all those in the Sham group survived (100%), and 70% of mice in the HCEPg group survived until the 5th day after sepsis inducation (Figure 2).

### 3.3. The Antimicrobial Properties of P. granatum Is Systemic

**Figure 3:**
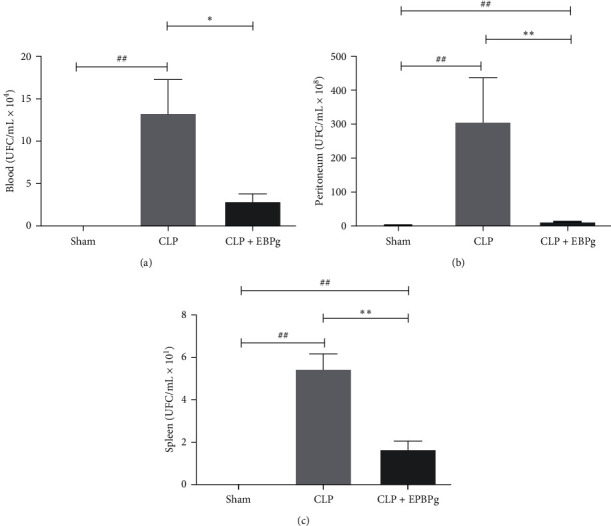
HCEPg reduced the number of colony-forming units (CFUs) in the peripheral blood (a), peritoneum (b), and spleen (c). The animals were divided into three groups: Sham (untreated Swiss mice without sepsis), CLP (untreated Swiss mice with sepsis by cecal ligation and puncture), and HCEPg (animals treated subcutaneously with a single dose of hydroalcoholic crude extract of the fruit peel (5 mg/kg) immediately after induction of sepsis). Data are presented as the mean ± SD (*n* = 5). ( ^*∗*^) *p* < 0.05 and ( ^*∗∗*^) *p* < 0.01 in comparison to the CLP group and (#) *p* < 0.05 and (##) *p* < 0.01 in comparison to the Sham group.

To continue investigating microbiological and physiological parameters, the experimental group with longer survival was monitored compared to Sham and CLP control mice. Treatment with HCEPg reduced the number of CFUs in blood (*p* = 0.0330) (see Figure 3(a)), peritoneum (*p* = 0.0079) (see Figure 3(b)) and spleen when compared with the CLP untreated group (*p* = 0.0079) (see Figure 3(c)).

### 3.4. Treatment with HCEPg Increases Cell Recruitment to the Peritoneal Cavity

**Figure 4:**
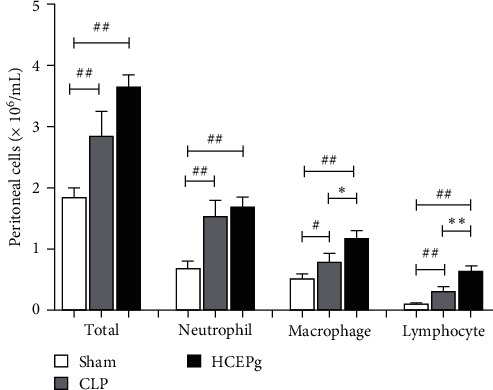
Treatment with *P. granatum* (HCEPg) increased cell recruitment in the peritoneum. The animals were divided into three groups (*n* = 5): Sham (untreated animals without sepsis), CLP (untreated animals with sepsis), and HCEPg (animals treated subcutaneously with a single dose of hydroalcoholic crude extract of the fruit peel (5 mg/kg)). ( ^*∗*^) *p* < 0.05 and ( ^*∗∗*^) *p* < 0.01 in comparison to the CLP group and (#) *p* < 0.05 and (##) *p* < 0.01 in comparison to the Sham group.

HCEPg treatment increased the number of macrophages (*p* = 0.0365 and *p* < 0.0001) and lymphocytes (*p* = 0.0055 and *p* < 0.0001) in the peritoneal cavity compared to control (CLP) and Sham groups, as well as the total number (*p* < 0.0001) of cells in the group treated with HCPEg in relation to Sham group. Regarding the number of neutrophils, there was an increase both in the CLP group and in the HCEPg group (see Figure 4).

### 3.5. *P. granatum* Reduces Lung Bleeding

HCEPg treatment was found to significantly reduce hemorrhage in the lung. No significant difference was observed between groups in the other evaluated parameters (see Table 1 and Figure S1).

### 3.6. HCEPg Activates T Helper and Dendritic Cells

**Figure 5:**
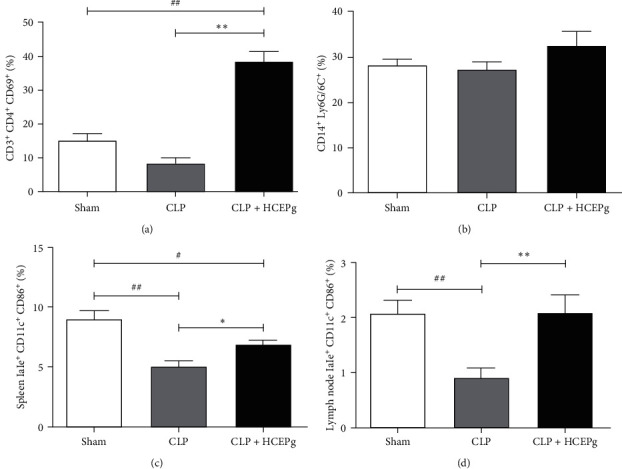
HCEPg activates immune cells in the spleen and peritoneum. The animals were divided into three groups: Sham (animals that underwent surgery but without cecum perforation and without treatment), CLP (untreated animals with sepsis induced by ligature and cecal perforation), and HCEPg (treated animals with sepsis induced by ligature and cecal perforation). Percentage of (a) activated CD4^+^ lymphocytes, (b) CD14/Ly6G/6C^+^ macrophages in the peritoneal cavity, (c) activated dendritic cells IaIe/CD11c/CD86^+^ in the spleen, and (d) activated dendritic cells IaIe/CD11c/CD86^+^ in the lymph node. ( ^*∗*^) *p* < 0.05 and ( ^*∗∗*^) *p* < 0.01 in comparison to the CLP group and (#) *p* < 0.05 and (##) *p* < 0.01 in comparison to the Sham group. The data represent the mean ± SD of five animals/group.

HCEPg increased the frequency of activated peritoneal T helper lymphocytes (*p* = 0.0001) (see Figure 5(a)) but had no effects on macrophages (see Figure 5(b)) in comparison to the CLP group. The numbers of activated dendritic cells (DCs) (CD11c^+^CD86^+^IaIe^+^) were augmented in spleen (*p* = 0.0412) (see Figure 5(c)) and lymph nodes (*p* = 0.0059) (see Figure 5(d)) obtained from HCEPg-treated animals in comparison with the CLP group. (Figure S2–S6).

We also investigated TCD8^+^CD69^+^ lymphocytes, but there was no statistical difference between CLP (17.62 ± 3.707) and CLP HCEPg (4.63 ± 5.948).

### 3.7. Treatment with HCEPg Decreases Peritoneal H_2_O_2_ Levels

**Figure 6:**
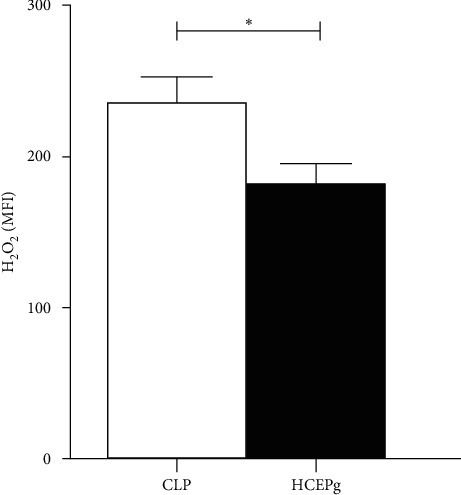
HCEPg decreases the production of hydrogen peroxide by peritoneal cells. The animals were divided into three groups: Sham (animals that received surgery but without cecum perforation and without treatment), CLP (untreated animals with sepsis by cecal ligation and perforation), and HCEPg (animals with sepsis induced by CLP and treated with HCEPg). Mean of the intensity of fluorescence in cells labeled with DHR. ( ^*∗*^) in relation to the CLP group, *p* < 0.05 in relation to the CLP group. The data represent the mean ± SD of five animals per group.

H_2_O_2_ secretion was lower in the HCEPg group than in the CLP group seeing the MFI (*p* = 0.0305) (see Figure 6), indicating a control of H_2_O_2_ production when there are large stimuli.

### 3.8. *P. granatum* Increases the Release of Cytokines

**Figure 7:**
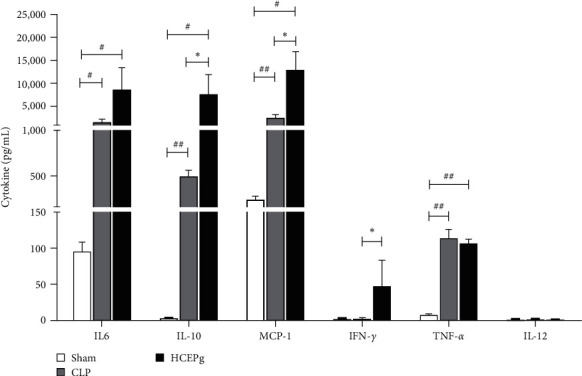
Serum cytokines were increased by HCEPg in sepsis mice. The animals were divided into three groups: Sham (animals that underwent surgery but without cecum perforation and without treatment), CLP (untreated animals with sepsis induced by ligature and cecal perforation), and HCEPg (treated animals with sepsis induced by ligature and cecal perforation). After serum collection, IL-6, IL-10, MCP-1, IFN-*γ*, TNF-*α*, and IL-12 were evaluated. ( ^*∗*^) *p* < 0.05 and ( ^*∗∗*^) *p* < 0.01 in comparison to the CLP group and (#) *p* < 0.05 and (##) *p* < 0.01 in comparison to the Sham group. The data represent the mean ± SD of five animals/group.

Considering that the treatment with *P. granatum* modulated phagocyte responses at the site of infection and that the evolution of sepsis occurs in parallel to systemic inflammation, we investigated cytokines related to the progression of sepsis and phagocyte function. *P. granatum* increased the production of IL-10 (*p* = 0.0159), MCP-1 (*p* = 0.0357), and IFN-*γ* (*p* = 0.0357). IL-6 (*p* = 0.0357), and TNF-*α* (*p* = 0.0079) compared to Sham group. IL-12 was not detected in any of the groups (see Figure 7).

## 4. Discussion

A single dose of the crude *P. granatum* peel extract HCEPg modulated the immune system by activating phagocytes and lymphocytes at the site of infection, controlling inflammatory responses by increasing IL-10 production and regulating H_2_O_2_ release by peritoneal cells [28]. The antimicrobial [21], anti-inflammatory [15], and antioxidant [29] properties of *P. granatum* reported previously, as well as the immunomodulatory action reported in the present study, contribute to the increased survival of animals with polymicrobial sepsis.

Two studies have been described in the literature with nonlethal sepsis by the CLP model in rats. In one of the studies, the rats were treated with the liquid extract of the fruit residue and had no positive effect on maintaining the survival of the animals nor on controlling the bacterial load [30]. In the other study, the rats were treated with the standardized fruit extract, resulting in anti-inflammatory and antioxidant effects on acute liver injury [13]. Therefore, our study with the standardized extract of the fruit peel in the lethal sepsis model is promising and innovative because HCEPg modulated the immune response contributing to the maintenance of survival of treated mice.

Evidence has shown that the antimicrobial activity of *P. granatum* is due to the presence of important phytochemical components in the crude extract, such as polyphenols (tannins and flavonoids), particularly punicalin, pedunculagin, and punicalagin [16]. Dahham et al. [31] evaluated the antimicrobial activity of several parts of *P. granatum* against Gram-negative and Gram-positive bacteria, as well as fungi, and demonstrated that the fruit peel presents the best antimicrobial activity. This was also observed in an *in vitro* study, in which *P. granatum* exhibited antimicrobial activity against enteropathogenic bacteria [18]. The antibacterial activity of *P. granatum* may be related to polyphenol structures as they may affect the bacterial cell wall, inhibit enzymes by oxidized agents, interact with proteins, and disturb the coaggregation of microorganisms [32–35].

In our study, *P. granatum* exhibited antimicrobial activity against gut bacteria *in vivo*. In the CLP model, the extract reduced bacterial proliferation at the infection site (peritoneal cavity) and in the blood and spleen, preventing dissemination to distant tissues such as the lungs, an important organ that is susceptible to acute lung injury characterized as a pathological inflammatory process in septic conditions, resulting in apoptosis of the epithelial cells [36, 37].

Another important phagocyte found in the peritoneal cavity is the macrophage, which promotes the maintenance of intestinal homeostasis and regulates host–microbiota symbiosis. Macrophages control the abundance of bacteria and fungi, limit the absorption of microbial molecules, and maintain intestinal integrity. The most severe sepsis occurs in septic mice with macrophage depletion and has been related to fecal *Candida* overgrowth, fecal pathogenic bacteria, intestinal barrier damage, and intestinal LPS and (1 ⟶ 3)-*β*-D-glucan translocation [38].

Cells from animals treated with *P. granatum* maintained H_2_O_2_ production but at lower levels than those in the CLP group. Controlling the release of reactive oxygen species is relevant for avoiding microbial proliferation without generating tissue damage and therefore contributes to restoring immune homeostasis in septic animals [39].

To verify the effect of HCEPg on secondary lymphoid organs and at the site of infection, we analyzed the cellular phenotype in the spleen, mesenteric lymph nodes, and peritoneum. In the spleen and mesenteric lymph nodes, treatment increased the activation of antigen-presenting cells, especially DCs. During sepsis, DCs reached maturation early in the immune response. *P. granatum* treatment accentuated DC activation and increased the capacity of those cells to act as antigen-presenting cells [40].

In our study, the number of DCs in the treated animals was higher than that of the CLP group, suggesting the role of the natural product in preventing cell mortality. Previous studies have shown that a profound loss of CD11c^+^ DCs in the spleen and lymph nodes is observed in untreated septic individuals due to the induction of caspase 3-dependent apoptosis in the first 12–36 hr after the development of sepsis [41–43].

Despite the increase in inflammatory cytokines (IL-6, IFN-*γ*) and chemokine (MCP-1), elevated concentrations of IL-10 were detected, which functions as a temporal regulator of the transition from early reversible sepsis to the late phase of irreversible shock. IL-6 can be secreted by macrophages in response to pathogen-associated molecular patterns elevated in patients with sepsis [44, 45], indicating that it is associated with the development of this condition [45]. Our results showed that IL-6 levels were maintained due to polymicrobial infection. MCP-1 is an important mediator of monocyte chemoattraction, and in our study, there was an increase of macrophage in the peritoneal cavity in treated animals compared to CLP animals; these cells are important in controlling microorganisms at the focus of infection [46].

Th1 cells, cytotoxic T cells, and natural killer cells produce IFN-*γ* [47]. These cells are closely associated with the activation and recruitment of other leukocytes, stimulating the elimination of infectious agents [48]. Schulte et al. [49] also confirmed that plasma IFN-*γ* levels are not directly related to severity or mortality in patients with sepsis.

During infection, the release of large quantities of inflammatory cytokines controls pathogen proliferation. However, an exacerbated inflammatory response can cause cell and tissue damage, worsening the clinical manifestations of the disease as observed in sepsis.

In addition, treatment of septic animals with HCEPg induced an increase in the CD4^+^ CD69^+^ T lymphocyte population, which is impaired in septic pictures with the significant reduction in the number of CD4 T cells, which affects the host response to infection [50–52]. In murine models of sepsis and postmortem tissue samples from septic patients, apoptosis has a major impact on immune cell depletion [42, 50, 53–55]. Herein, TCD8^+^ lymphocytes were investigated, but no increase in this population was found in the treated animals.

A limitation of the study was the lack of investigation of the activity of isolated components from the fruit peel extract, such as punicalagin, as previous data indicate its potential as an antimicrobial and anti-inflammatory compound. Nonetheless, the data presented herein, highlights the importance of *P. granatum* peel extract as a modulator of sepsis-induced responses in the host. This has now been discussed in the appropriate session.

## 5. Conclusions

Sepsis treatment is highly challenging as each patient responds very differently to medical interventions, and there is no universally accepted treatment for sepsis. Patients, in general, have a highly dysregulated immune system, ranging from a state of excessive inflammation to a state of immunosuppression. Herein, we show that HCEPg may be a potential treatment for lethal polymicrobial sepsis. The extract acts directly by inhibiting the growth and proliferation of infectious microorganisms and indirectly by modulating the immune system and controlling the inflammatory response. These two characteristics are ideal for drugs used in sepsis treatment, as many currently used antibiotics are only active against microorganisms and do not promote the restoration of immune homeostasis in patients with sepsis.

## Figures and Tables

**Table 1:** Effect of the hydroalcoholic extract of the *P. granatum* fruit peel on the lung inflammation during the sepsis by cecal ligation and puncture.

Group	Hemorrhage	Infiltrated	Necrosis	Edema
Sham^a^	2.0 ± 1.1^b^	1.5 ± 0.5	1 ± 1.1	2.0 ± 1.1
CLP	3.5 ± 0.5	2.5 ± 0.5	1.5 ± 0.5	2.0 ± 1.1
HCEPg	2.0 ± 0.1 ^*∗*^	2.0 ± 0.1	1.7 ± 0.5	2.0 ± 0

^a^Sham, untreated animals without sepsis; CLP, untreated animals with sepsis and HCEPg, animals treated subcutaneously with a single dose of hydroalcoholic crude extract of the fruit peel (5 mg/kg). ^b^Values are expressed as mean ± standard deviation. Kruskal–Wallis non-parametric ANOVA and Student's *t*-test. ( ^*∗*^) *p* < 0.05 and ( ^*∗∗*^) *p* < 0.01 in comparison to the CLP group and (#) *p* < 0.05 and (##) *p* < 0.01 in comparison to the Sham group.

## Data Availability

The data used to support the findings of this study are included in this article.

## Supplementary Materials

Supplementary Materials Histological analysis of the lung of septic animals from the Sham, CLP, and HCEPg groups (Figure S1). Strategies for the acquisition of cell populations by immunophenotyping from the Sham, CLP, and HCEPg groups (Figures S2–S6).
