# Supplementary material for: The Immunomodulatory Activity of Punica granatum L. Peel Extract Increases the Lifespan of Mice with Lethal Sepsis
Source: J Immunol Res. 2023 Aug 16;2023:2868707. doi: 10.1155/2023/2868707 (PMC10447006; doi:10.1155/2023/2868707)
Supplement: Supplementary Materials — Histological analysis of the lung of septic animals from the Sham, CLP, and HCEPg groups (Figure S1). Strategies for the acquisition of cell populations by immunophenotyping from the Sham, CLP, and HCEPg groups (Figures S2–S6). [file 2868707.f1.pdf]

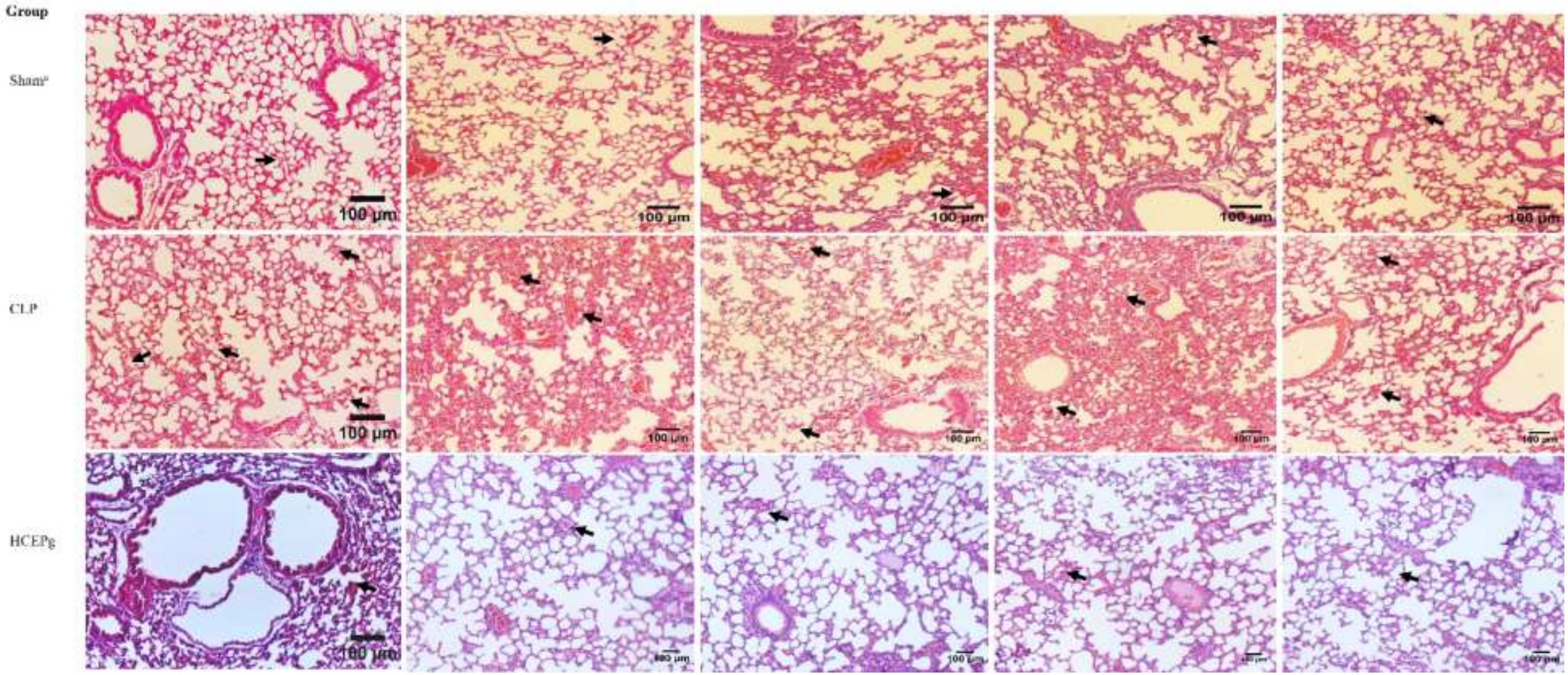

Supplement figure 1: **Histological analysis of hemorrhage in the lung of septic mice from the Sham, CLP and HCEPg groups.** The animals received, subcutaneously PBS (CLP) or HCEPg (5 mg/kg) just after infection. The lungs were removed and fixed in 10% formalin. The Sham group presented normal alveolar architecture, while the CLP group presented hemorrhage in the alveoli; the HCEPg group presented normal alveolar structure. Histological sections stained by H&E, hematoxylin and eosin. The arrows in the images point to the hemorrhage in the lung of the groups. Scale bar is 100µm.

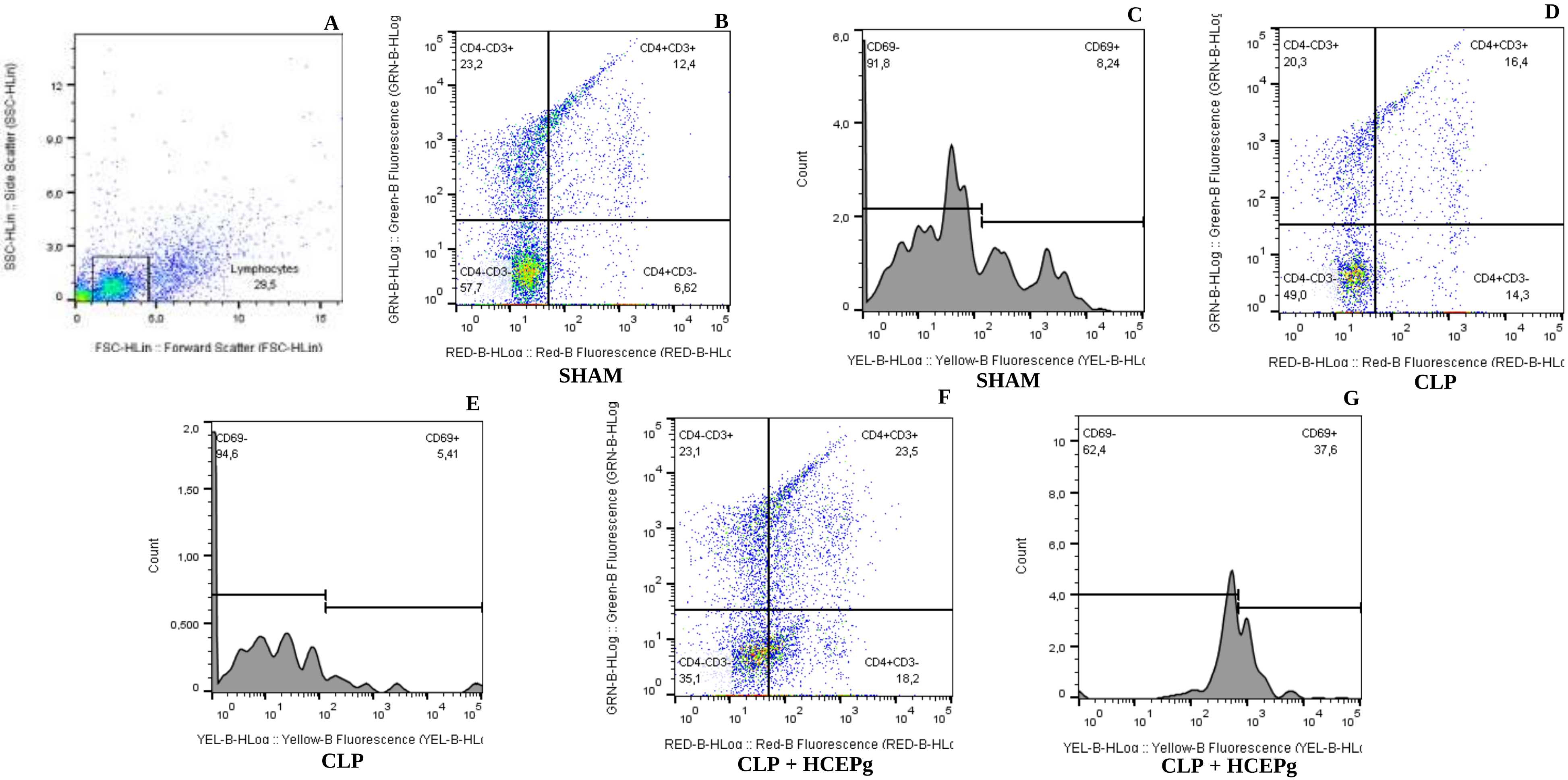

Supplement figure 2: **Acquisition strategies of the lymphocyte population and activated lymphocytes in the peritoneum by immunophenotyping.** (A) Acquisition gate of the lymphocyte (CD3$^+$) population for subsequent acquisition of lymphocyte subpopulations. (B) Acquisition gate of the population of double-labeled CD3$^+$CD4$^+$ cells from the SHAM group. (C) Representative histogram of the acquisition of the CD3$^+$CD4$^+$ CD69$^+$ population indicating activated lymphocytes in the SHAM group. (D) Acquisition gate of the population of double-labeled CD3$^+$CD4$^+$ cells from the CLP group. (E) Histogram representing the acquisition of the CD3$^+$CD4$^+$ CD69$^+$ population indicating activated lymphocytes in the CLP group. (F) Gate of acquisition of the population of double-labeled CD3$^+$CD4$^+$ cells from the CLP+HCEPg group. (G) Representative histogram of the acquisition of the CD3$^+$CD4$^+$ CD69$^+$ population indicating activated lymphocytes in the CLP+HCEPg group.

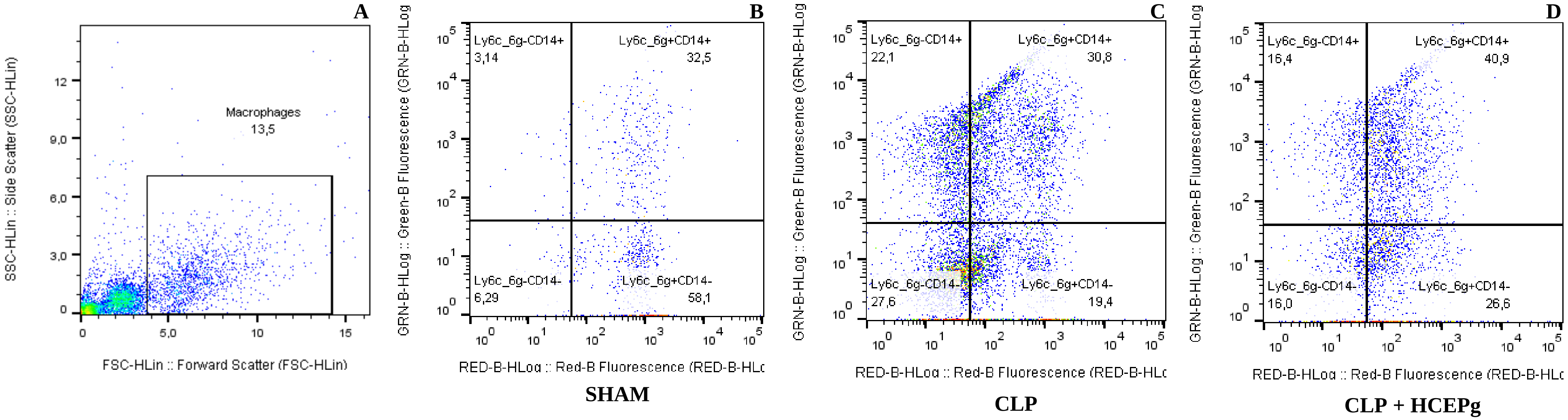

Supplement figure 3: **Acquisition strategies of the phagocyte population in the peritoneum by immunophenotyping.** (A) Acquisition gate of the phagocyte population ($CD14^+$). (B) Acquisition gate of the double-tagged $CD14^+Ly6C/6G^+$ cell population of the SHAM group. (C) Acquisition gate of the double-tagged $CD14^+Ly6C/6G^+$ cell population of the CLP group. (D) Acquisition gate of the population of double-tagged $CD14^+Ly6C/6G^+$ cells from the CLP+HCEPg group. Macrophages were defined as $CD14^+Ly6C/6G^+$ and neutrophils as $CD14\text{-}Ly6C/6G^+$.

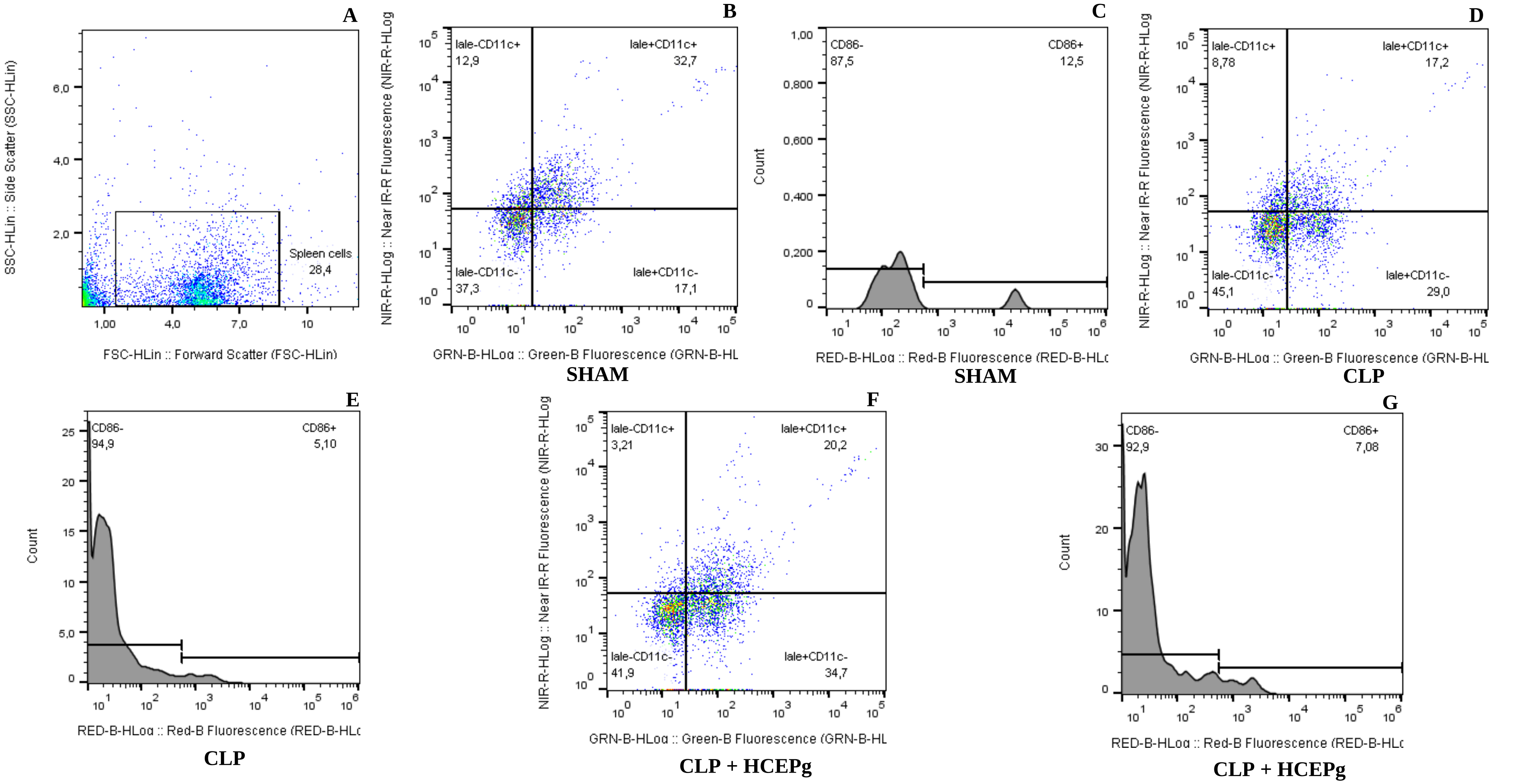

Supplement figure 4: **Strategies for acquisition of the population of dendritic cells and activated dendritic cells in the spleen by immunophenotyping.** Spleen cells were arranged by size (FSC) versus IaIe expression to define the population of antigen presenting cells (A). To delineate the subpopulation of dendritic cells, CD11c^{+} marker was used and to observe the degree of activation of this subpopulation, CD86 marker expression was used. (B) Gate acquisition of the double-labeled IaIe^{+}CD11c^{+} cell population from the SHAM group. (C) Representative acquisition histogram of the IaIe^{+}CD11c^{+}CD86^{+} population indicating activated dendritic cells in the spleen in the SHAM group. (D) Acquisition gate of the double-tagged IaIe^{+}CD11c^{+} cell population from the CLP group. (E) Histogram representing the acquisition of the IaIe^{+}CD11c^{+}CD86^{+} population indicating activated dendritic cells in the spleen in the CLP group. (F) Gate acquisition of the IaIe^{+}CD11c^{+} double-labeled cell population in the CLP+HCEPg group. (G) Representative histogram of the acquisition of the IaIe^{+}CD11c^{+}CD86^{+} population indicating activated dendritic cells in the spleen in the CLP+HCEPg group.

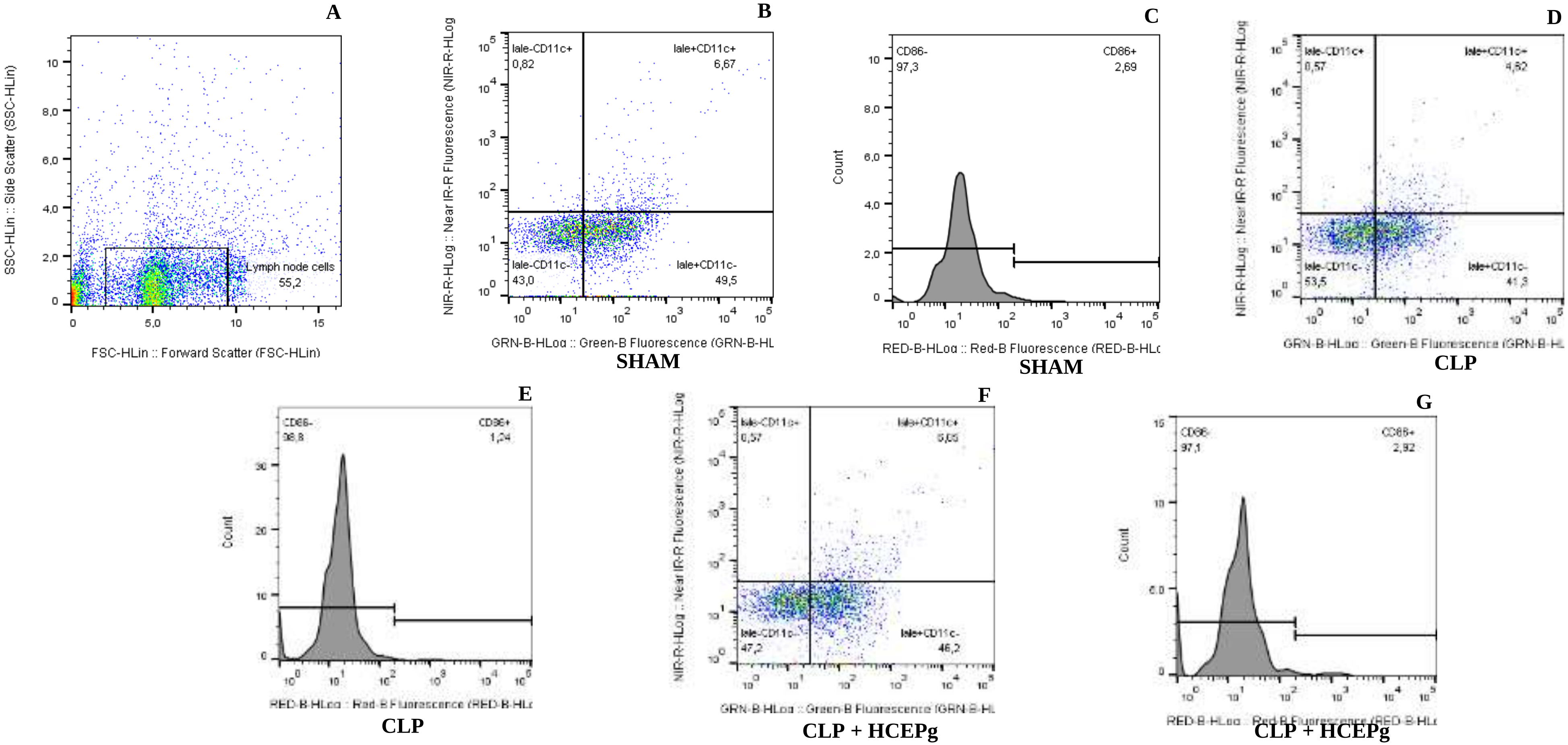

Supplement figure 5: **Strategies for acquisition of the population of dendritic cells and activated dendritic cells in the lymph node by immunophenotyping.** Lymph node cells were arranged by size (FSC) versus IaIe expression to define the population of antigen presenting cells (A). To delineate the subpopulation of dendritic cells, CD11c$^+$ marker was used and to observe the degree of activation of this subpopulation, CD86 marker expression was used. (B) Gate acquisition of the population of double-labeled IaIe$^+$CD11c$^+$ cells from the SHAM group. (C) Representative acquisition histogram of the IaIe$^+$CD11c$^+$CD86$^+$ population indicating activated dendritic cells in the lymph node in the SHAM group. (D) Acquisition gate of the double-labeled IaIe$^+$CD11c$^+$ cell population from the CLP group. (E) Histogram representing the acquisition of the IaIe$^+$CD11c$^+$CD86$^+$ population indicating activated dendritic cells in the lymph node in the CLP group. (F) Gate acquisition of the IaIe$^+$CD11c$^+$ double-labeled cell population in the CLP+HCEPg group. (G) Representative histogram of the acquisition of the IaIe$^+$CD11c$^+$CD86$^+$ population indicating activated dendritic cells in the lymph node in the CLP+HCEPg group.

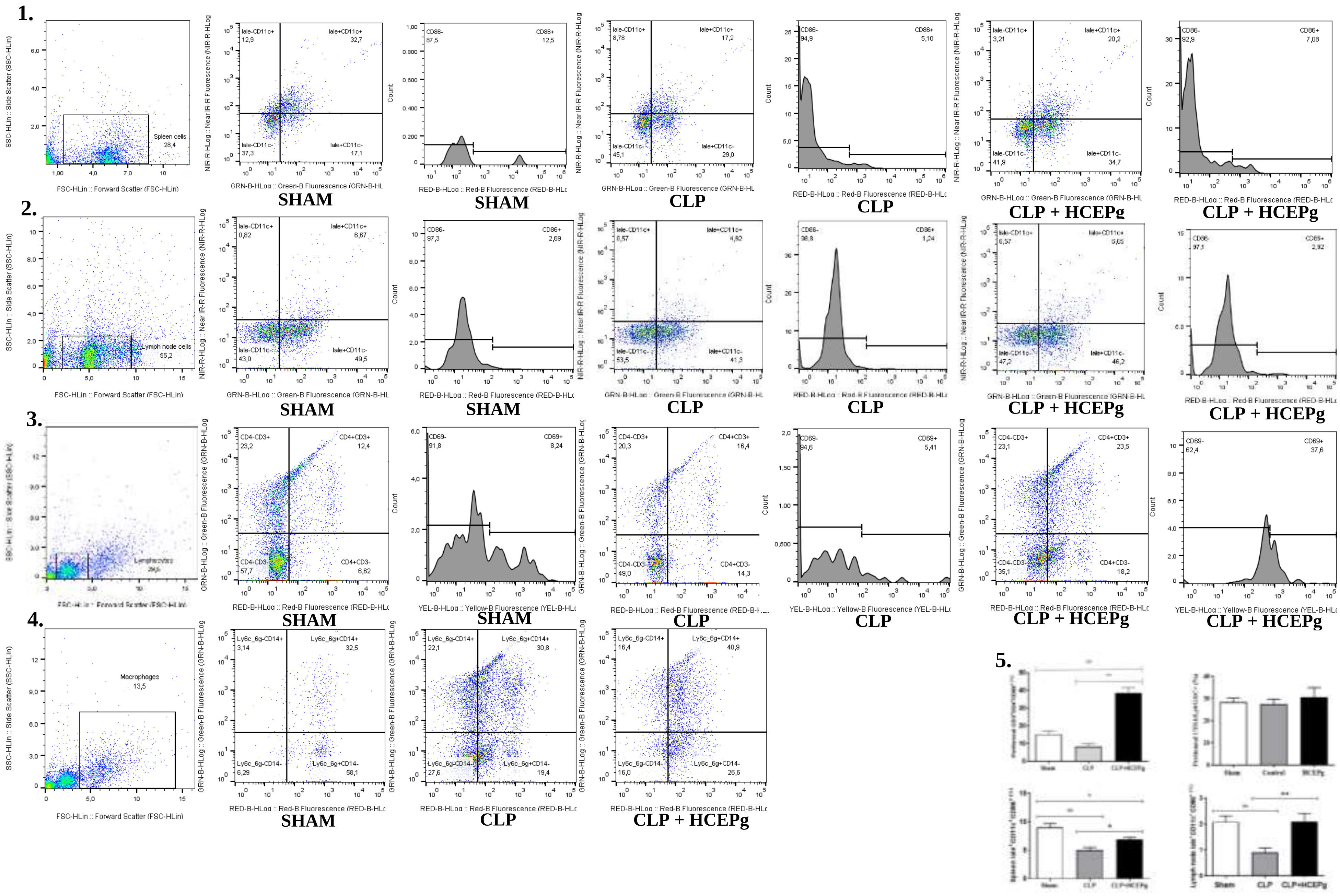

1.
Spleen cells
28,4
FSC-HLin :: Forward Scatter (FSC-HLin)
SSC-HLin :: Side Scatter (SSC-HLin)
Iale-CD11c+ 12,9
Iale+CD11c+ 32,7
Iale-CD11c- 37,3
Iale+CD11c- 17,1
GRN-B-HLog :: Green-B Fluorescence (GRN-B-HL
NIR-R-HLog :: Near IR-R Fluorescence (NIR-R-HLog
SHAM
CD86- 87,5
CD86+ 12,5
Count
RED-B-HLog :: Red-B Fluorescence (RED-B-HL
SHAM
Iale-CD11c+ 8,78
Iale+CD11c+ 17,2
Iale-CD11c- 45,1
Iale+CD11c- 29,0
CLP
CD86- 94,9
CD86+ 5,10
CLP
Iale-CD11c+ 3,21
Iale+CD11c+ 20,2
Iale-CD11c- 41,9
Iale+CD11c- 34,7
CLP + HCEPg
CD86- 92,9
CD86+ 7,08
CLP + HCEPg
2.
Lymph node cells
55,2
Iale-CD11c+ 0,82
Iale+CD11c+ 6,67
Iale-CD11c- 43,0
Iale+CD11c- 49,5
SHAM
CD86- 97,3
CD86+ 2,69
SHAM
Iale-CD11c+ 0,57
Iale+CD11c+ 4,62
Iale-CD11c- 53,5
Iale+CD11c- 41,3
CLP
CLP
CLP + HCEPg
CD86- 97,1
CD86+ 2,92
CLP + HCEPg
3.
Lymphocytes
CD4-CD3+ 23,2
CD4+CD3+ 12,4
CD4-CD3- 57,7
CD4+CD3- 6,62
RED-B-HLog :: Red-B Fluorescence (RED-B-HL
GRN-B-HLog :: Green-B Fluorescence (GRN-B-HLog
SHAM
CD69- 91,8
CD69+ 8,24
YEL-B-HLog :: Yellow-B Fluorescence (YEL-B-HL
SHAM
CD4-CD3+ 20,3
CD4+CD3+ 16,4
CD4-CD3- 49,0
CD4+CD3- 14,3
CLP
CD69- 94,6
CD69+ 5,41
CLP
CD4-CD3+ 23,1
CD4+CD3+ 23,5
CD4-CD3- 35,1
CD4+CD3- 18,2
CLP + HCEPg
CD69- 62,4
CD69+ 37,6
CLP + HCEPg
4.
Macrophages
13,5
Ly6c_6g-CD14+ 3,14
Ly6c_6g+CD14+ 32,5
Ly6c_6g-CD14- 6,29
Ly6c_6g+CD14- 58,1
SHAM
Ly6c_6g-CD14+ 22,1
Ly6c_6g+CD14+ 30,8
Ly6c_6g-CD14- 27,6
Ly6c_6g+CD14- 19,4
CLP
Ly6c_6g-CD14+ 16,4
Ly6c_6g+CD14+ 40,9
Ly6c_6g-CD14- 16,0
Ly6c_6g+CD14- 26,6
CLP + HCEPg
5.
Sham
CLP
CLP+HCEPg

Supplement figure 6: **Acquisition strategies of dendritic cell population and activated dendritic cells by immunophenotyping.** (Line 1) Spleen cells were arranged by size (FSC) versus IaIe expression to define the population of antigen presenting cells. The following acquisitions refer to the populations of $IaIe^{+}CD11c^{+}$ double-labeled dendritic cells and $IaIe^{+}CD11c^{+}CD86^{+}$ activated dendritic cells of the respective groups. (Line 2) Acquisitions for antigen-presenting cell population in the lymph node following the same strategy used in the spleen. (Line 3) Acquisition of lymphocyte population cells in the peritoneum. The following acquisitions refer to the population of doubly labeled $CD3^{+}CD4^{+}$ cells and subsequently the subpopulation of activated $CD3^{+}CD4^{+}$ $CD69^{+}$ lymphocytes for the respective groups. (Line 4) Acquisition of cells from the phagocyte population in the peritoneum. The following acquisitions refer to the $CD14^{+}Ly6C/6G^{+}$ double-labeled cell populations of the respective groups. (5) Representative graph of the percentage of activation of lymphocytes (a), macrophages in the peritoneal cavity (b), dendritic cells in the spleen (c) and lymph node (d).
